# Application value of metagenomic next-generation sequencing in hematological patients with high-risk febrile neutropenia

**DOI:** 10.3389/fcimb.2024.1366908

**Published:** 2024-04-25

**Authors:** Xiao Wang, Huiye Zhang, Nan Zhang, Shan Zhang, Yanrong Shuai, Xiaojuan Miao, Yilan Liu, Ling Qiu, Shihui Ren, Sihan Lai, Ying Han, Hao Yao, Xupai Zhang, Fangyi Fan, Haoping Sun, Hai Yi

**Affiliations:** ^1^ Department of Hematology, The General Hospital of Western Theater Command, Chengdu, China; ^2^ School of Pharmacy, Chengdu Medical College, Chengdu, China; ^3^ Department of Pharmacy, Chengdu Eighth People’s Hospital, Chengdu, China

**Keywords:** metagenomic next-generation sequencing, febrile neutropenia, infection, pathogen diagnosis, cell-free DNA

## Abstract

**Background:**

Metagenomic next-generation sequencing (mNGS) is a novel non-invasive and comprehensive technique for etiological diagnosis of infectious diseases. However, its practical significance has been seldom reported in the context of hematological patients with high-risk febrile neutropenia, a unique patient group characterized by neutropenia and compromised immune responses.

**Methods:**

This retrospective study evaluated the results of plasma cfDNA sequencing in 164 hematological patients with high-risk febrile neutropenia. We assessed the diagnostic efficacy and clinical impact of mNGS, comparing it with conventional microbiological tests.

**Results:**

mNGS identified 68 different pathogens in 111 patients, whereas conventional methods detected only 17 pathogen types in 36 patients. mNGS exhibited a significantly higher positive detection rate than conventional methods (67.7% *vs*. 22.0%, *P* < 0.001). This improvement was consistent across bacterial (30.5% *vs*. 9.1%), fungal (19.5% *vs*. 4.3%), and viral (37.2% *vs*. 9.1%) infections (*P* < 0.001 for all comparisons). The anti-infective treatment strategies were adjusted for 51.2% (84/164) of the patients based on the mNGS results.

**Conclusions:**

mNGS of plasma cfDNA offers substantial promise for the early detection of pathogens and the timely optimization of anti-infective therapies in hematological patients with high-risk febrile neutropenia.

## Introduction

Febrile neutropenia (FN) is the most prevalent complication for hematological patients. It is estimated that over 80% of patients will develop FN as a result of the underlying disease, chemotherapy, high-dose radiotherapy, immunotherapy, and transplantation (Keng and Sekeres, 2013). FN represents a significant burden to patients as it predisposes them to serious and recurring life-threatening infections, delays or reduces the intensity of chemotherapy treatment, and potentially shortens overall survival (Klastersky et al., 2016; Boccia et al., 2022). The timely identification of causative pathogens and immediate administration of appropriate anti-infective treatment is of major importance in reducing mortality and improving the quality of life (Kuderer et al., 2006). However, the clinical symptoms of FN are often non-specific, with most patients only experiencing a fever (Freifeld et al., 2011; Schmidt-Hieber et al., 2019). Moreover, conventional microbiological tests (CMT) have a low efficiency and positivity rate in detecting pathogens, with less than 15% of FN patients being documented by blood cultures (Blennow et al., 2014; Feng et al., 2023). The pathogens and infection sites are also unclear. Therefore, there is still a lack of satisfactory detection methods for the rapid, accurate, and efficient identification of pathogens in FN patients.

Metagenomic next-generation sequencing (mNGS) is a promising, hypothesis-free diagnostic approach that has the potential to directly detect common pathogens (Blauwkamp et al., 2019; Geng et al., 2021). Compared with conventional methods, mNGS has higher sensitivity and faster detection times (Grumaz et al., 2016). Additionally, it has a unique advantage in identifying mixed and rare pathogen infections (Doan et al., 2016; Chen et al., 2021; Gu et al., 2021; Deng et al., 2023). In recent years, an increasing amount of research has demonstrated the great potential of mNGS in diagnosing clinical infectious diseases in hematological patients with FN (Guo et al., 2022; Zhang et al., 2022). However, the sample sizes of these studies are too small, and the results are controversial. Additionally, research on the impact of treatment outcomes and antibiotic administration using mNGS has been extremely limited. Furthermore, there is a lack of studies specifically targeting high-risk FN patients. The true significance of mNGS in hematological patients with high-risk FN remains unclear. Therefore, we conducted a retrospective study to assess the diagnostic performance of mNGS and its clinical impact on antimicrobial therapy, compared with conventional methods, in high-risk FN patients.

## Methods

### Study patients

We performed a retrospective analysis of patient records who underwent a plasma mNGS test for suspected infections at the General Hospital of Western Theater Command in Chengdu, China, from October 2020 to December 2022. The Institutional Review Board and Ethics Committee of the Hospital approved this study. The approval number of the ethics committee is 2023EC5-ky087. Given the retrospective nature of the study, the need for informed written consent was waived. The inclusion criteria for the patients were as follows: (1) Diagnosis of a hematological disease; (2) Age over 18 years; (3) High-risk FN; (4) If the patient has received empirical antibiotic treatment but is currently showing poor efficacy (a decrease in peak fever of < 0.5°C or no significant change after receiving empirical treatment for 72∼96 hours); (5) The patient underwent conventional microorganism testing and mNGS testing simultaneously. Hematologic diseases mainly include leukemia, lymphoma, multiple myeloma, aplastic anemia, etc. High-risk FN patients had to fulfill the following criteria: (1) Absolute neutrophil count (ANC) < 500/μL; (2) Single oral temperature ≥ 38.3°C (axillary temperature ≥ 38.0°C), or twice consecutive oral temperature ≥ 38.0°C (axillary temperature ≥ 37.7°C for ≥ 1 h); (3) Anticipated prolonged and severe neutropenia (ANC < 100/μL) lasting for more than 7 days and/or significant medical co-morbid conditions, including hypotension, pneumonia, newly developed abdominal pain, or neurological changes (Averbuch et al., 2013; Chinese Society of Hematology, 2020). The exclusion criteria included: (1) Primary immunodeficiency or human immunodeficiency virus (HIV); (2) Fevers caused by medication or underlying diseases; (3) Incomplete medical records; (4) A clear diagnosis of infectious etiology at the time of enrollment.

### CMT and mNGS

Peripheral blood samples were collected from the same site on the day of inclusion (D0) and sent for conventional microorganism testing and mNGS testing at the same time. Conventional microorganism tests consisted of blood cultures, polymerase chain reaction (PCR) tests (for CMV, EBV, HHV1, etc.), and sterile tissue smear microscopy or culture, with each culture lasting for a duration of 5 days. All the mNGS testing was undertaken by Genoxor Medical Science and Technology Inc., as outlined in a previous study (Xu et al., 2022). Additional details on mNGS and bioinformatics analysis can be found in the **Supplementary Methods**. Only DNA-based pathogens were identified through mNGS. In real-time, the results of both conventional methods and mNGS were made available to the primary physician as soon as possible. Although the mNGS test was performed only once on D0, conventional microorganism tests were carried out multiple times based on clinical necessity. Simultaneously, procalcitonin tests, high-sensitivity C-reactive protein (CRP) assays, (1,3)-β-D-glucan tests (G tests), galactomannan tests (GM tests), T-SPOT, inflammatory cytokines, radiological examinations, and ultrasonic examinations were conducted multiple times, also depending on clinical need.

### Clinical adjudication

If the pathogens detected were not commonly reported, the mNGS results were interpreted in the context of the patients’ clinical features. Otherwise, those detected reads were categorized as sequences of non-pathogenic microbes. A Clinical Expert Committee was created, which included a hematologist, a clinical microbiologist, and a radiologist. The clinical committee retrospectively evaluated the presence of infection, taking into account conventional microorganism tests and other clinical data. This data included laboratory findings, imaging and ultrasound results, and patient symptoms, among others. Prior to identifying the pathogen, all patients were administered empirical anti-infective treatment following the Chinese guidelines for the clinical application of antibacterial drugs for agranulocytosis with fever (2020) (Chinese Society of Hematology, 2020). Adjustments to the subsequent anti-infective treatments were made primarily based on the evaluation of treatment effectiveness, results of microbiological tests, and other conventional tests. Conventional methods were deemed to yield positive results if pathogens were identified by conventional microorganism tests between D0 and day D7.

### Statistical analysis

Statistical analysis and graphic presentation were carried out using SPSS 18.0 software (IBM), GraphPad Prism 8.0 (GraphPad Software), WPS Office software. Continuous variables were represented by medians and ranges, while categorical variables were indicated by counts and percentages. To compare categorical variables, we used either the Chi-square test or Fisher’s exact test. For continuous variables comparison, the Mann–Whitney U test was deployed. A 2×2 contingency table was set up to calculate sensitivity, specificity, positive predictive value (PPV), and negative predictive value (NPV). A *P* value < 0.05 denoted statistical significance.

## Results

### Patient characteristics

The study group consisted of 164 participants (**Supplementary Figure 1**). Patient characteristics were summarized in **Table 1**. The median age was 33 years (range 18∼70), with males comprising 69.5% (114/164) of the cohort. Acute myeloid leukemia (AML) and acute lymphoblastic leukemia (ALL) were the most common underlying diagnoses seen in 45.7% (75/164) and 28.0% (46/164) of subjects, respectively. Neutropenia, largely caused by chemotherapy, was observed in 62.2% (102/164) of the patients. Hematopoietic stem cell transplantation (HSCT) had been performed on 22.6% (37/164) of the participants, and chimeric antigen receptor T-cell therapy was administered in 11.0% (18/164) of cases. Elevated serum procalcitonin levels reaching or exceeding 0.5 ng/L were seen in 38.4% (63/164) of the participants, and high sensitivity C-reactive protein levels ≥ 50 mg/L were detected in 74.4% (122/164) of cases at Day 0. A vast majority, 92.1% (151/164), of participants had peripherally inserted central catheters or central venous catheters. Prior to mNGS tests, empiric anti-infective treatment was administered in 82.3% (135/164) of cases. Based on clinical diagnoses, patients were categorized into either infected group (140 cases, 85.4%) or non-infected group (24 cases, 14.6%). Pneumonia was the predominant infectious complication, presenting in 40.9% (64/164) of cases. Diagnostic criteria for sepsis and septic shock were met by 35.4% (56/164) and 22.6% (37/164) of participants, respectively. Patients maintained a median number of 4 (range 2∼8) blood cultures between Day 0 and Day 7. The in-hospital mortality rate within 28 days was 14.0% (23/164).

**Table 1:** Clinical characteristics of enrolled patients in this study.

Characteristics	Patients Enrolled (n=164)
Age (years), range	18∼70
Median (IQR)	33 (24∼45)
Sex, n (%)	
Male	114 (69.5%)
Female	50 (30.5%)
Underlying diseases, n (%)	
Acute myeloid leukemia	75 (45.7%)
Acute lymphocytic leukemia	46 (28.0%)
Lymphoma	15 (9.1%)
Aplastic anemia	9 (5.5%)
Myelodysplastic syndrome	7 (4.3%)
Other disease	12 (7.3%)
Therapy of underlying diseases, n (%)	
Chemotherapy	102 (62.2%)
Hematopoietic stem cell transplantation	37 (22.6%)
Chimeric antigen receptor T-cell therapy	18 (11.0%)
Other therapy	7 (4.3%)
Procalcitonin	
PCT < 0.5 ng/mL	101 (61.6%)
PCT ≥ 0.5 ng/mL	63 (38.4%)
High-sensitivity C-reactive protein	
HS-CRP < 50 mg/L	42(25.6%)
HS-CRP ≥ 50 mg/L	122(74.4%)
Exposure to anti-infective agents prior to mNGS, n (%)	135 (82.3%)
Venous catheter indwelling, n (%)	151 (92.1%)
Number of blood culture, range	2-8
Median (IQR)	4 (3-5)
Pneumonia, n (%)	67 (40.9%)
Sepsis, n (%)	58 (35.4%)
Septic shock, n (%)	37 (22.6%)
Death, n (%)	23 (14.0%)

IQR, interquartile range; PCT, procalcitonin; HS-CRP, high-sensitivity C-reactive protein.

### Pathogens by mNGS and conventional methods

**Figure 1:**
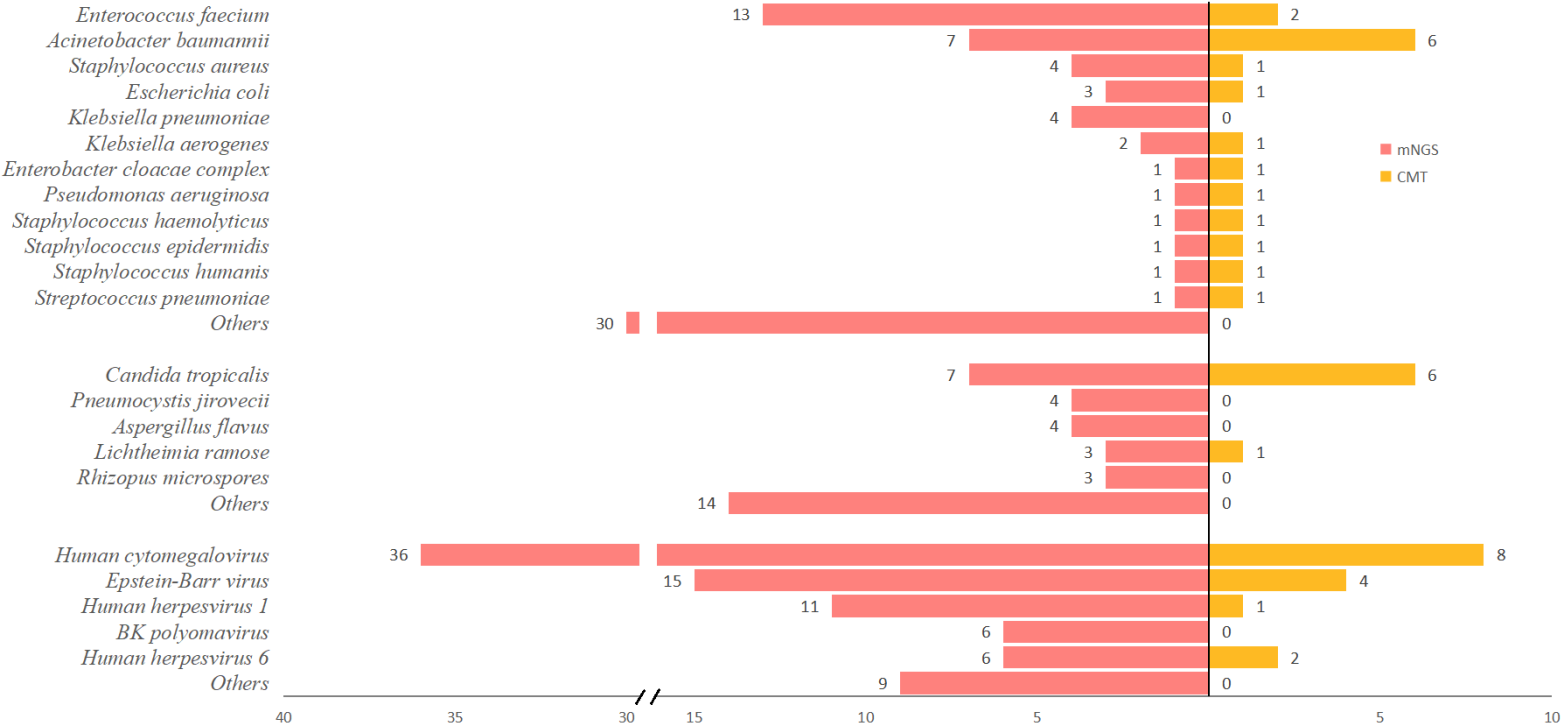
Pathogenic microorganisms detected by mNGS and CMT.

**Figure 1** showcases the most common pathogenic spectrum of infections detected by mNGS and conventional methods. In this study, mNGS successfully identified 68 different types of pathogens among 111 patients. The bacteria most frequently detected by mNGS included *Enterococcus faecium*, *Acinetobacter baumannii*, *Staphylococcus aureus*, *Escherichia coli*, and *Klebsiella pneumoniae*. Moreover, the most common fungi were *Candida tropicalis*, *Aspergillus flavus*, *Pneumocystis jirovecii*, *Lichtheimia ramose*, and *Rhizopus microspores*. As for viruses, the most frequently identified were Human cytomegalovirus, Epstein-Barr virus, Human herpesvirus 1, Human herpesvirus 6, and BK polyomavirus.

The conventional methods identified 17 types of pathogens across 36 patients. Out of these, bacterial cultures were positively identified in only 23 patients. The bacteria most commonly identified by these methods included *Acinetobacter baumannii*, *Escherichia coli*, *Enterococcus faecium*, *Klebsiella pneumoniae*, and *Staphylococcus aureus*. *Candida tropicalis* was the most common fungus identified, accounting for 85.7% (6/7) of detected fungi. A single case of mucormycosis was confirmed through a pathological examination. The viruses most often detected encompassed Human cytomegalovirus, Epstein-Barr virus, Human herpesvirus 1, and Human herpesvirus 6.

### Comparison of mNGS and conventional methods

**Figure 2:**
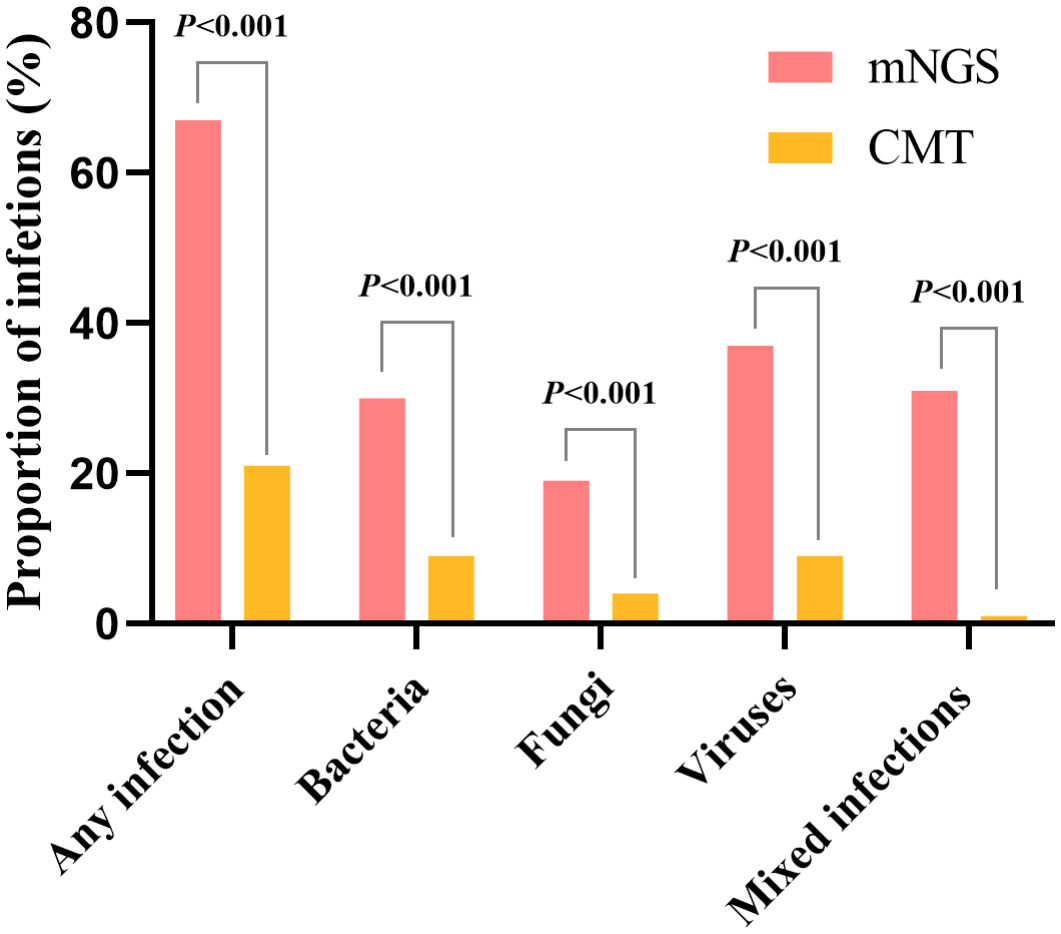
Proportions of different types of infections.

Electronic reports of mNGS results were usually dispatched to clinicians within 24 h for more than half of the patients studied. The median pathogen detection time with mNGS was significantly quicker than that with conventional methods: 24 h (range 24∼72) *vs*. 96 h (range 24∼168), respectively (P < 0.001). Furthermore, mNGS demonstrated a significantly greater positive detection rate than conventional methods (67.7% *vs*. 22.0%, P < 0.001) (**Figure 2**). This held true for bacterial (30.5% *vs*. 9.1%) and fungal (19.5% *vs*. 4.3%) species and viruses (37.2% *vs*. 9.1%) (P < 0.001 for each comparison). It is also important to note that mNGS was markedly more effective in identifying mixed infections than conventional methods (31.7% *vs*. 1.2%, P < 0.001). In individual patients, mNGS detected up to four pathogens, whereas conventional methods could only detect a maximum of two.

**Figure 3:**
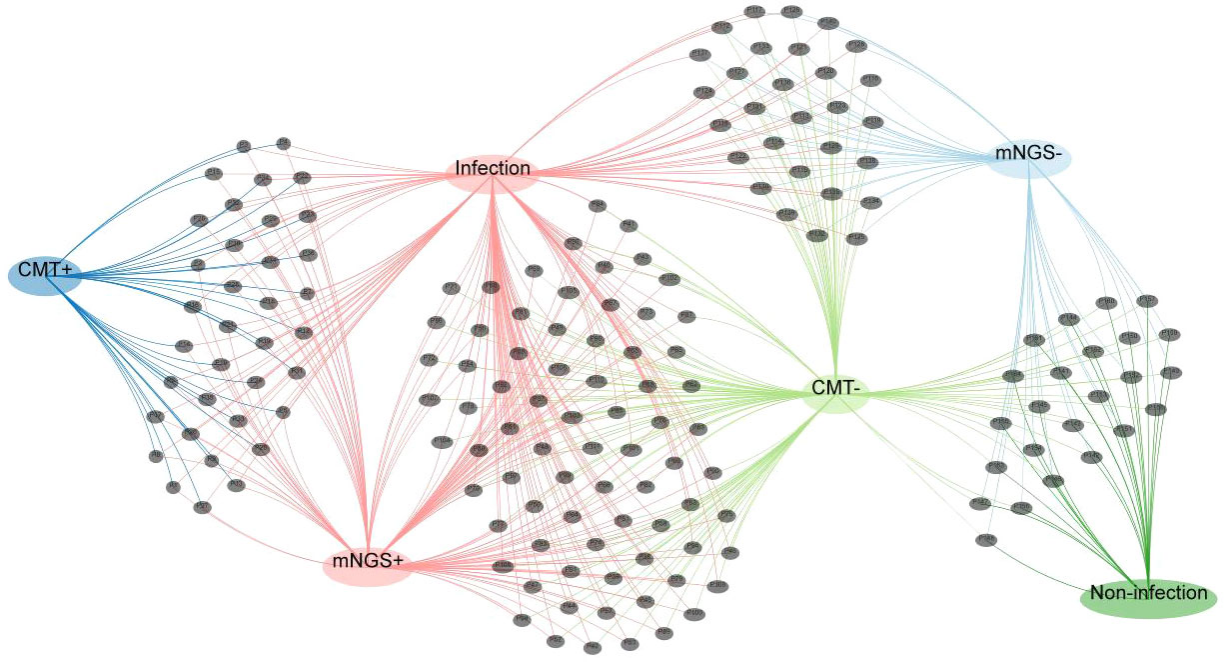
Venn network diagram depicting the number of infections. Each black dot represents a febrile neutropenia patient.

The consistency between mNGS and conventional methods was also explored (**Figure 3**). When both tests produced positive or negative results, we considered the results to be consistent. In 54.3% (89/164) of the cases, concordant results were observed, while discordant results were noticed in 45.7% (75/164) of the participants. In 22.0% (36/164) of cases, both mNGS and conventional methods produced positive results, whereas in 32.3% (53/164) of participants, the results were negative in both instances. For 45.7% (75/164) of the participants, mNGS yielded positive results, whereas conventional methods indicated negative results. All pathogens identified by conventional methods were also detected through mNGS. In cases where mNGS results were negative, the conventional methods also correspondingly yielded negative results.

The diagnostic accuracy of mNGS was remarkably superior to that of conventional methods (82.3% *vs*. 36.6%, P < 0.001). The sensitivity, specificity, positive predictive value (PPV), and negative predictive value (NPV) of mNGS and conventional methods were depicted in **Supplementary Table 1**. mNGS demonstrated a three-fold increase in sensitivity (79.3% *vs*. 25.7%, P < 0.001), compared with conventional methods. Specificity did not differ between the two groups.

### Clinical impact of plasma mNGS

The anti-infective treatment plans for 51.2% (84/164) of the participants were adjusted mainly in accordance with the mNGS results (**Table 2**). In addition, 9.8% (16/164) of the participants underwent a change in their antimicrobial spectrum, and 11.0% (18/164) started receiving de-escalation therapy. In 22.6% (37/164) of cases, at least one anti-infective agent was discontinued, of which the usage of all anti-infective agents was entirely ceased in 16 instances. Additionally, at least one anti-infective agent was introduced in the treatment plans for 7.9% (13/164) of the participants.

**Table 2:** Clinical impact of plasma mNGS.

Anti-infective Treatment	Patients (n=164)
Any change	84 (51.2%)
Changing antimicrobial spectrum	16 (9.8%)
Antimicrobial de-escalation	18 (11.0%)
Removing ≥ 1 anti-infective agent	37 (22.6%)
Removing all anti-infective agent	16 (9.8%)
Adding ≥ 1 anti-infective agent	13 (7.9%)

## Discussion

Febrile neutropenia is a common complication in patients with hematologic diseases and is associated with high morbidity and mortality (Rosa and Goldani, 2014). Most patients with FN will not have either an identifiable clinical focus of infection or positive cultures (Escrihuela-Vidal et al., 2019). High-risk febrile neutropenia patients have an even higher mortality rate. Clinicians usually prescribe broad-spectrum antibiotics initially and often maintain antibiotic treatment until recovery from neutropenia. Inappropriate empirical antibiotic therapy can lead to increased bacterial resistance, adverse drug reactions, longer hospital stays, higher economic burdens, and increased mortality (Martinez-Nadal et al., 2020). Moreover, overuse of antibiotics can reduce gut microbiota diversity and increase the risk of gastrointestinal graft-versus-host disease in allogeneic hematopoietic cell transplant recipients (Holler et al., 2014). Consequently, timely and accurate pathogen identification is crucial for appropriate antibiotic use.

In clinical practice, we commonly use biomarkers, such as procalcitonin, CRP, (1-3)-β-D-glucan, and galactomannan for the diagnosis of infections (Guo et al., 2010; Lu et al., 2011a; Wacker et al., 2013; Li et al., 2015). However, these biomarkers cannot definitively identify the infecting pathogen. CMTs typically include pathogen culture, nucleic acid amplification tests, serological tests, and microscopic examination. Pathogen culture can identify the type of pathogens, but it has a low positivity rate and is time-consuming. The PCR method and serological tests can also be used to identify pathogens, but it requires a prior prediction of the possible types of pathogens (Lu et al., 2011b). Microscopic examination is often unfeasible in FN patients owing to an unclear clinical focus of infection and the difficulty in collecting samples.

mNGS presents a rapid, non-culture-based, and unbiased method, which mainly avoids the limitations of traditional diagnostics. Ever since its first application in diagnosing neuroleptospirosis in 2014, mNGS has gained widespread recognition in scientific research and clinical practice (Wilson et al., 2014). Although numerous studies have confirmed the efficacy of mNGS in enhancing pathogen detection among FN patients, most have only compared it with traditional methods at a single time point, thus failing to accurately reflect clinical practices (Benamu et al., 2022; Schulz et al., 2022). Typically, owing to cost concerns, mNGS testing is performed only once, whereas traditional methods can involve multiple tests. In most instances, an mNGS test is administered only to high-risk FN patients, particularly when conventional microbial cultures turn up negative, empirical treatments are ineffective, or the medical case is complex and multifaceted. Therefore, in this study, we evaluated the clinical utility of mNGS by comparing it with multiple conventional tests in high-risk FN patients. We aimed to gauge the applicability of mNGS in a setting that mirrors real-world clinical scenarios more closely.

Our study highlighted several significant benefits of mNGS in high-risk FN patients. First, compared with the conventional methods, mNGS demonstrated remarkably higher sensitivity and clinical agreement in detecting potential pathogens. The specificity of mNGS showed no significant difference, compared with the conventional methods. Moreover, we found that the mNGS positive rate seems less likely to be influenced by prior antimicrobial use. Despite numerous microorganism culture tests, the positive rate remained low. The outcomes from conventional methods were linked to previous antimicrobial use. These observations aligned closely with prior studies (Feng et al., 2023). The mNGS method was able to detect nearly all pathogens identified by traditional methods. If a pathogen could not be detected by the NGS method, it was unlikely to be identified by conventional methods.

Second, mNGS technology uncovered a broader spectrum of infectious pathogens in FN patients. It detected a total of 68 pathogens, compared with the 17 identified by the conventional methods. However, there was a considerable overlap in the identification of the most commonly detected pathogens between mNGS and conventional methods. Many pathogens detected by mNGS, such as *Aspergillus flavus, Pneumocystis jirovecii*, and BK polyomavirus, were never detected by conventional methods in our department. However, the clinical manifestations of patients were consistent with the results of mNGS. The mNGS methods also showcased significant advantages in detecting fungal infections, viral infections, and most importantly, mixed pathogen infections. It could identify bacteria, fungi, and viruses in a single test, while the positive detection rate for fungi and viruses is notably low with conventional testing (Jerome et al., 2019). FN patients with weakened immune systems are particularly vulnerable to fungal and viral infections. Without available evidence, fungi and viruses are rarely identified as the cause of early fever in FN (Boeriu et al., 2022). Traditional methods for detecting fungal and viral infections are limited and often lead to missed diagnoses. Our results affirmed that mNGS could detect more fungi and viruses. Moreover, the detection rate of mixed pathogen infections by mNGS was higher than that by conventional methods. We even detected four pathogens in a single mNGS test, including two bacteria, one fungus, and one virus. Rarely could conventional methods detect more than two pathogens concurrently. The mNGS method identified many rare pathogens, such as *Nocardia*, *Fusarium*, and *Mucor*. The bacterium most commonly detected by NGS was the gram-positive *Enterococcus faecium*, and infections by gram-positive bacteria were slightly more prevalent. This contrasts with previous studies, which indicated the most identified bacteria were *Escherichia coli*, and infections by gram-negative bacteria were significantly more prevalent (Feng et al., 2023; Xu et al., 2023). This discrepancy may be due to the fact that 82.3% of patients in our study received empiric anti-infective therapy prior to NGS testing, with the majority being treated with carbapenem antibiotics.

Third, we found that the mNGS method serves as an alternative non-invasive diagnostic tool for focal infection pathogens. It detects the DNA fragments of pathogens emanating from the focal point of infection, rather than the living pathogens themselves. This is particularly beneficial for FN patients who, owing to thrombocytopenia, often struggle to tolerate invasive procedures, such as bronchoalveolar lavage and aspiration biopsy. Additionally, invasive procedures carry risks of developing complications like hypoxemia or endobronchial bleeding (Choo et al., 2019). Our research indicated the relatively high success rate of plasma mNGS tests in diagnosing pulmonary infections.

Fourth, mNGS offers a considerably faster diagnostic process, compared with conventional methods (24 h *vs.* 96 h), thereby facilitating prompt clinical decision-making regarding diagnosis and treatment, especially with respect to adjusting antimicrobial therapy. Therefore, the consequences of delayed treatment are minimized, thereby enhancing the potential for successful interventions.

Fifth, mNGS positively influences antimicrobial adjustments. It is widely recognized that the unnecessary use of antibiotics can contribute to the development of drug resistance. In this regard, mNGS has the potential to reduce the unnecessary prescription of antibiotics. In this study, 51.2% of all patients made adjustment to their antibiotics regimen, with 33.5% of patients predominantly de-escalating or reducing antibiotics based on mNGS results. This observation was similar to the report of previous studies (Xu et al., 2023). The mortality rate of patients whose anti-infective treatment was adjusted according to mNGS was comparable with that of previous patients. No evidence was found to suggest that adjusting antibiotics regimen led to poor prognosis. Consequently, mNGS may offer valuable evidence for adjusting antibiotics regimen, potentially providing further benefits by preventing the overuse of antibiotics. Because this was a retrospective study and no randomization was performed, we did not further compare other factors, such as the total amount of antibiotics used, duration of antibiotic use, and adverse effects of drugs. In subsequent studies, we plan to further explore the impact of mNGS on the use of anti-infective drugs and prognosis through randomized controlled trials.

Finally, implementing mNGS on a large scale could be feasible and cost-effective. Typically, the mNGS test is conducted only once owing to its high cost. However, with the declining price of mNGS, patients could potentially benefit from repeated testing over time. Currently, the cost of mNGS testing is under $200. Generally, optimizing the use of antibiotics based on mNGS results can reduce healthcare expenses related to antibiotics overuse. For instance, the cost of voriconazole, the most common antifungal agent for FN patients, over 2 days actually exceeds that of an mNGS test. Therefore, if the mNGS test results suggest that voriconazole could be discontinued 2 days earlier, both medical expenses and healthcare resources could be significantly reduced.

Our study had several limitations. First, this is a retrospective study, with many NGS tests performed following the negative results of conventional methods and the ineffectiveness of empirical treatment, which affected the positive detection rate of conventional methods. Second, there is still a lack of rigorous standards for distinguishing whether a pathogen detected by mNGS is genuinely pathogenic or merely colonizing. Pathogens identified by mNGS have not undergone nucleic acid amplification tests for molecular verification. Moreover, it remains challenging to completely eliminate transient contaminations originating from the sampling procedure or laboratory environment. Consequently, the final outcome is often determined by the clinician’s subjective experience. This fact also contributed to the observed high specificity, and there is a possibility of false positives in the mNGS tests. Third, diagnosing infections and adjusting antibiotics regimen are inherently subjective processes often influenced by the interpretations of the treating physicians. Fourth, a low DNA load of pathogens might result in false-negative outcomes (Purhonen et al., 2015). For example, the positive detection rate of *Mycobacterium tuberculosis* is often lower than the actual infection rate because it is difficult to extract its DNA. Finally, we only conducted testing for DNA pathogens and did not test for RNA viruses, which may have led to potential underdiagnosis.

In conclusion, our research indicates that mNGS of plasma cfDNA holds significant potential for pathogen detection, especially in the diagnosis of fungal infections, which enables early optimization of anti-infective therapy in high-risk FN patients. This will facilitate the establishment of mNGS-based anti-infection guidelines in the future, which can further clarify the timing for initiating and discontinuing anti-infection treatments and make the use of anti-infection drugs more rational and evidence-based. Ultimately, it can reduce the use of antibiotics, curb the rise of drug resistance, decrease drug-related adverse reactions, and improve patient prognosis. Despite these potential advantages, mNGS is currently not the typical method for microbiological diagnosis in all settings. Conventional methods remain prevalent in many clinical laboratories owing to their user-friendly nature, low cost, and capacity to deliver quick results under particular conditions. However, with continuous improvements and wider accessibility of sequencing technologies, mNGS may gradually become a more commonplace tool for microbiological diagnosis. The combined use of mNGS and conventional tests may pave the way for the most effective diagnostic strategy. However, before mNGS testing garners widespread use in clinical practice, it is imperative that we further assess its clinical value through larger, multicenter randomized controlled studies.

## Data availability statement

The raw data supporting the conclusions of this article will be made available by the authors, without undue reservation.

## Ethics statement

The studies involving humans were approved by The Institutional Review Board and Ethics Committee of the General Hospital of Western Theater Command. The studies were conducted in accordance with the local legislation and institutional requirements. Written informed consent for participation was not required from the participants or the participants’ legal guardians/next of kin because Given the retrospective nature of the study, the need for informed written consent was waived.

## Author contributions

XW: Conceptualization, Funding acquisition, Investigation, Methodology, Project administration, Validation, Writing – original draft. HZ: Conceptualization, Data curation, Funding acquisition, Writing – original draft. NZ: Conceptualization, Data curation, Project administration, Writing – original draft. SZ: Investigation, Methodology, Writing – review & editing. YS: Methodology, Writing – review & editing. XM: Investigation, Methodology, Validation, Writing – review & editing. YL: Investigation, Methodology, Writing – review & editing. LQ: Investigation, Methodology, Writing – review & editing. SR: Investigation, Methodology, Writing – review & editing. SL: Investigation, Methodology, Writing – review & editing. YH: Investigation, Methodology, Writing – review & editing. HYa: Methodology, Software, Writing – review & editing. XZ: Methodology, Software, Visualization, Writing – review & editing. FF: Methodology, Software, Validation, Writing – review & editing. HS: Conceptualization, Writing – original draft. HYi: Conceptualization, Project administration, Writing – original draft.

## Supplementary material

Supplementary Table 1 Diagnostic efficiency of mNGS and CMT. PPV, positive predictive value; NPV, negative predictive value.
