# Supplementary material for: Application value of metagenomic next-generation sequencing in hematological patients with high-risk febrile neutropenia
Source: Front Cell Infect Microbiol. 2024 Apr 25;14:1366908. doi: 10.3389/fcimb.2024.1366908 (PMC11079123; doi:10.3389/fcimb.2024.1366908)
Supplement: Supplementary Table 1 — Diagnostic efficiency of mNGS and CMT. PPV, positive predictive value; NPV, negative predictive value. [file Table_1.docx]

**Supplementary Table 1. Diagnostic efficiency of mNGS and CMT**

|  | **mNGS** | **CMT** | ***P*** |
| --- | --- | --- | --- |
| Sensitivity | 79.3% | 25.7% | <0.001 |
| Specificity | 100% | 100% | - |
| PPV | 100% | 100% | - |
| NPV | 45.3% | 18.8% | <0.001 |

PPV, positive predictive value; NPV, negative predictive value.
