# Supplementary material for: Application value of metagenomic next-generation sequencing in hematological patients with high-risk febrile neutropenia
Source: Front Cell Infect Microbiol. 2024 Apr 25;14:1366908. doi: 10.3389/fcimb.2024.1366908 (PMC11079123; doi:10.3389/fcimb.2024.1366908)
Supplement: Supplementary file 2 [file DataSheet_1.pdf]

Metagenomic next-generation sequencing (mNGS)

Blood samples taken from the patients were drawn into 10-mL Vacuumm blood collection tubes (Kang Jian, CHN). The whole blood was centrifuged at 1900g for 10 minutes to separate plasma. Subsequently, cf DNA was extracted using Magnetic Serum/ Plasma DNA Maxi Kit (TIANGEN, CHN) following the manufacturer's instructions. DNA extraction yield was quantified using a QuantiT dsDNA HS Assay Kit and Qubit 3.0 Fluorometer (Thermo Scientific, USA). The Nextera XT DNA Library Preparation Kit (Illumina, USA) was used for library construction, including end repair, adenylating, adapter ligation, and purification. The quality of the libraries was assessed by a 2100 Bioanalyzer using the High Sensitivity DNA Assay (Agilent Technologies, USA). The 75bp single-end sequencing was conducted in accordance with the instructions for the Illumina NextSeq 550 Dx sequencer. The samples as the No-Template Control (NTC) were sequenced simultaneously to assess contaminations during the wet-lab experiments.

Bioinformatics analysis of species-level abundance profiling

Raw sequencing data were first split using bcl2fastq2, and subjected to a quality control process for trimming adapter sequences and removing low-quality reads by Trimmomatic v0.36. Next, the reads mapping to the human reference genome GRCh37 were excluded using the short-read alignment tool Bowtie v2.2.6. Taxonomic classification of microbial reads was conducted using Kraken v2.0.9-beta and a custom k-mer database that was constructed using 51,543 genomes of ~27,000 species (14863 bacteria, 738 fungi, 11500 viruses, 118 parasites) from the NCBI assembly databases.
