# Supplementary material for: Application value of metagenomic next-generation sequencing in hematological patients with high-risk febrile neutropenia
Source: Front Cell Infect Microbiol. 2024 Apr 25;14:1366908. doi: 10.3389/fcimb.2024.1366908 (PMC11079123; doi:10.3389/fcimb.2024.1366908)
Supplement: Supplementary file 3 [file Image_1.pdf]

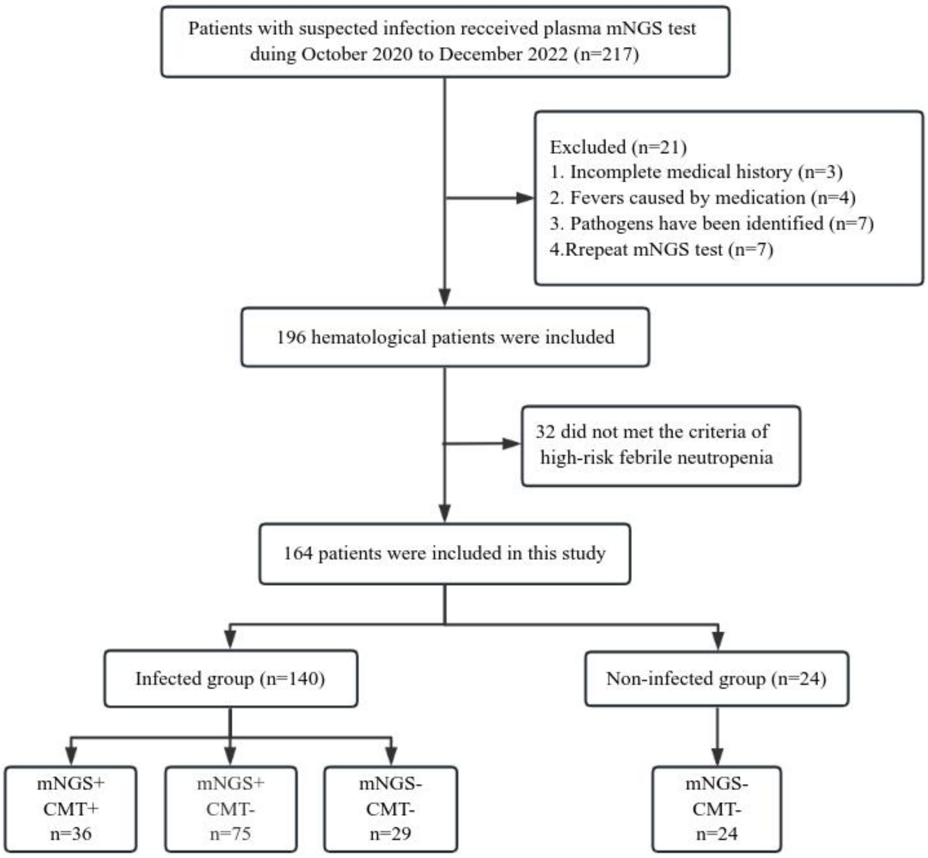


Supplementary Figure 1. A schematic of the study profile. CMT, conventional microbiological tests.
